# Pain Catastrophizing in Rheumatoid Arthritis and Spondyloarthritis Patients Receiving Biologic Therapy: A Cross-Sectional Study

**DOI:** 10.7759/cureus.97825

**Published:** 2025-11-26

**Authors:** El Kacem El Marbouh, Ihsane Hmamouchi, Laila Taoubane, Abdellah Ould Mohamed, Alhassane Alidou Garba, Houda El Assad, Wissal Belhadj, Najlae El Ouardi, Hamza Toufik, Abderrahim Majjad, Ahmed Bezza

**Affiliations:** 1 Rheumatology, Mohammed V Military Training Hospital, Rabat, MAR; 2 Clinical Epidemiology, Faculty of Medicine, Health Sciences Research Center (CReSS) International University of Rabat (UIR), Rabat, MAR

**Keywords:** anxiety, depression, pain catastrophizing, rheumatoid arthritis, spondyloarthritis

## Abstract

Pain catastrophizing is an important psychological factor influencing disease experience in inflammatory rheumatic diseases. Data on its prevalence and determinants in rheumatoid arthritis (RA) and spondyloarthritis (SpA) patients receiving biologic therapy remain scarce. In this cross-sectional study, 126 patients (86 RA, 40 SpA) under biologic therapy were evaluated. Sociodemographic, clinical, and comorbidity characteristics were recorded. Disease activity was assessed using the Disease Activity Score in 28 Joints using C-reactive Protein (DAS28-CRP), the Clinical Disease Activity Index (CDAI), the Simplified Disease Activity Index (SDAI), and the Health Assessment Questionnaire (HAQ) for RA, and the Ankylosing Spondylitis Disease Activity Score using C-reactive Protein (ASDAS-CRP), the Bath Ankylosing Spondylitis Disease Activity Index (BASDAI), and the Bath Ankylosing Spondylitis Functional Index (BASFI) for SpA. Psychological assessment included the Pain Catastrophizing Scale (PCS), the Generalized Anxiety Disorder-7 questionnaire (GAD-7), and the Patient Health Questionnaire-9 (PHQ-9). Logistic regression was performed to identify factors associated with catastrophizing (PCS >30). RA patients were older, predominantly female, and had more comorbidities compared with SpA patients. Catastrophizing was more prevalent in RA (52.3%) than in SpA (25%, p=0.004), with higher rumination (p=0.003) and helplessness (p<0.001). Anxiety (77.9% vs. 57.5%, p=0.018) and depression (83.7% vs. 55%, p<0.001) were also more frequent in RA. In univariate analysis, catastrophizing was associated with female sex, higher educational level, RA phenotype, anxiety, and depression, while pain intensity was inversely associated. In multivariate analysis, higher education (OR 7.76, p=0.035), anxiety (OR 6.87, p=0.004), and lower pain Value Added Scale (VAS; OR 0.52, p<0.001) remained independent predictors. Catastrophizing, anxiety, and depression are highly prevalent in RA patients compared with SpA. The paradoxical inverse association with pain intensity suggests catastrophizing reflects cognitive-emotional processes rather than nociceptive severity, underscoring the importance of systematic psychological assessment in biologically treated patients.

## Introduction

Chronic inflammatory rheumatic diseases (CIRDs), such as rheumatoid arthritis (RA) and spondyloarthritis (SpA), are progressive conditions that often require long-term treatment with disease-modifying antirheumatic drugs (DMARDs), including biologics. Early initiation of these therapies is crucial to reduce disease activity, prevent structural damage, and improve quality of life [1].

It is well established that RA predominantly affects older adults, whereas SpA tends to occur in younger individuals. This demographic difference may influence psychological responses to pain and therapy [2].

Pain catastrophizing is a cognitive-emotional response characterized by an exaggerated negative orientation toward pain, involving rumination, magnification, and feelings of helplessness [3]. It is associated with worse pain outcomes, increased disability, and poorer response to treatment [3]. According to the Diagnostic and Statistical Manual of Mental Disorders (DSM-5), depression is characterized by a depressed mood (sadness, emptiness, hopelessness) and/or a loss of interest or pleasure in nearly all activities, present most of the day, nearly every day, for at least two weeks [4]. Generalized anxiety disorder (GAD) is a persistent and often severe mental disorder of the anxiety spectrum, characterized by persistent (six months or more), excessive worrying, anxiety, tension associated with symptoms of hypervigilance, and other somatic symptoms of anxiety [5]. While depression and anxiety are well-documented comorbidities in CIRDs, the role of catastrophizing, particularly in patients receiving biologic therapy, remains understudied.

This study aimed to compare pain catastrophizing, depression, and anxiety scores in RA and SpA patients receiving biologic therapy and to identify factors associated with catastrophizing. The primary objective was to compare pain catastrophizing between RA and SpA patients receiving biologic therapy, and the secondary objectives were to compare anxiety and depression between the two groups and to identify clinical and psychological factors associated with catastrophizing. We hypothesized that RA patients would exhibit higher levels of catastrophizing compared to SpA patients, driven by differences in disease characteristics, age distribution, and psychological comorbidities.

## Materials and methods

Study design and patients

We conducted a cross-sectional study at the Rheumatology Service of the Mohammed V Military Training Hospital, Rabat, Morocco, between 16 July 2024 and 16 September 2024. All patients included in the study had a confirmed diagnosis of RA or SpA made by board-certified rheumatologists, according to the American College of Rheumatology (ACR)/European League Against Rheumatism (EULAR) criteria and spondyloarthritis (SpA) according to the Assessment of Spondyloarthritis International Society (ASAS) classification criteria for axial/peripheral SpA [6,7].

Patients were consecutively recruited during routine intravenous biologic infusions or prescription-renewal visits at the rheumatology outpatient clinic of a tertiary university hospital. They received biologic therapies, including anti-TNF, anti-IL-6, anti-CD20, or JAK inhibitors, administered subcutaneously or intravenously according to standard clinical protocols.

Inclusion and exclusion criteria

Patients were eligible for inclusion if they were aged 18 years or older and had a confirmed diagnosis of RA or SpA according to the ACR/EULAR or ASAS classification criteria. All participants were required to have been receiving biologic therapy for at least six months prior to enrollment.

Patients with active psychiatric disorders necessitating psychiatric intervention (e.g., major depressive disorder, psychotic disorders, or generalized anxiety disorder requiring pharmacologic treatment) were excluded. Participants with subclinical or mild-to-moderate symptoms of anxiety or depression were included.

Ethical approval

All participants provided written informed consent in accordance with the Declaration of Helsinki. The Ethics Committee for Biomedical Research, Mohammed V University issued approval (approval number: 88/25).

Questionnaire and clinical assessment

We collected sociodemographic data from the patients, including age, sex, and educational level, as well as clinical data such as disease duration, comorbidities (diabetes, hypertension, heart disease, dyslipidemia, thyroid disorders, smoking, uveitis, chronic kidney disease, inflammatory bowel disease, and psoriasis), type of biologic therapy received, and duration of exposure. A history of depression was recorded from patient self-report and/or medical chart review. Pain intensity was assessed using a 0-10 Visual Analogue Scale (VAS), where 0 indicates no pain, and 10 indicates the worst imaginable pain [8].

In RA, disease activity was assessed using the Disease Activity Score in 28 Joints using C-reactive Protein (DAS28-CRP), the Clinical Disease Activity Index (CDAI), and the Simplified Disease Activity Index (SDAI), while functional status was evaluated with the Health Assessment Questionnaire (HAQ) [9-11]. In SpA, disease activity was measured using the Ankylosing Spondylitis Disease Activity Score using C-reactive Protein (ASDAS-CRP) and the Bath Ankylosing Spondylitis Disease Activity Index (BASDAI), and functional impairment was assessed with the Bath Ankylosing Spondylitis Functional Index (BASFI) [12-14].

The Arabic version of the Pain Catastrophizing Scale (PCS) is a validated 13-item questionnaire that assesses catastrophic thinking related to pain [15]. The PCS is available for free academic use for students, physicians, clinical practice, and non-funded academic users. We can access the questionnaire and its available translations directly. It was used for non-commercial, academic purposes in accordance with fair use principles. Participants reflected on past painful experiences and indicated how often each of the 13 thoughts or feelings occurred on a scale from 0 (not at all) to 4 (all the time). The PCS includes three subscores: Rumination: Items 8-11; Magnification: Items 6, 7, 12; Helplessness: Items 1-5, 12. A total score ≥30 indicates high catastrophizing.

The Arabic version of the Patient Health Questionnaire-9 (PHQ-9) was used to assess depression severity [16,17]. Patients rated the frequency of depressive symptoms over the past four weeks on a scale from 0 (not at all) to 3 (nearly every day); a total score ≥ 5 indicates a mild depression.

The Arabic version of the GAD-7 scale was used to assess anxiety severity [16,17]. Patients rated the frequency of anxiety symptoms over the past four weeks on a scale from 0 (not at all) to 3 (nearly every day); a total score ≥5 indicates mild anxiety.

Certain potential confounders, including pain chronicity and fibromyalgia, were not systematically evaluated due to the focus on primary variables and data availability. Detailed descriptions of the questionnaires (in Arabic and English) and scoring methods are provided in Appendix A.

Data analysis

Descriptive statistics were used to summarize the data. Continuous variables were expressed as mean ± standard deviation (SD) if normally distributed or median (interquartile range (IQR)) if non-normally distributed. Categorical variables were expressed as frequencies and percentages.

Statistical analyses were conducted using Student’s t-test for continuous parametric variables, the Mann-Whitney U test for continuous non-parametric variables, and the chi-square or Fisher’s exact test for categorical variables.

Variables presented in the tables were categorized as continuous (age, BMI, disease duration, VAS, DAS28-CRP, CDAI, SDAI, ASDAS-CRP, BASDAI, BASFI, HAQ) or categorical (sex, educational level, comorbidities, type of biologic therapy, history of depression/anxiety). Appropriate statistical tests were selected according to variable type, as described above. Multiple logistic regression analyses were conducted to assess multivariate associations between PCS scores and potential predictors (e.g., depression, anxiety).

All questionnaires were fully completed, with no missing data. Patients were reminded to complete any initially skipped items, ensuring responses remained unbiased, so no imputation was necessary.

Statistical analyses were performed using Jamovi software (version 2.7.4.0; The jamovi project (2023). retrieved from https://www.jamovi.org).

Sample size calculation

A sample size of 126 patients (63 per group) was calculated to achieve 80% power with α=0.05 (two-tailed), based on an expected difference in PCS scores between RA and SpA patients. The effect size was estimated from previous studies [18,19].

## Results

A total of 126 patients were included, comprising 86 with RA and 40 with SpA. Compared with SpA patients, those with RA were significantly older (59.2 ± 13.1 vs. 49.4 ± 11.5 years, p < 0.001) and more frequently female (89.5% vs. 35.5%, p < p<0.001) and exhibited higher levels of pain (3.4 ± 1.5 vs. 1.7 ± 1.1, p < 0.001). Educational level also differed significantly, with illiteracy more frequent in RA patients (56.9% vs. 5%, p < 0.001). No significant differences were observed in housing or BMI. Comorbidities were more prevalent in the RA group, including hypertension (23.2% vs. 7.5%, p=0.033), diabetes (12.7% vs. 0%, p=0.018), and dyslipidemia (18.6% vs. 0%, p=0.004), whereas uveitis was reported exclusively in SpA patients (27.5%, p<0.001) (Table 1).

**Table 1:** Patient characteristics Categorical variables ¶ are presented as n (%) and compared using the chi-square test. Continuous variables are expressed as mean±standard deviation δ or median and interquartile interval (IQR) (§) and compared using the t-test or the Mann-Whitney U test. The p-value considered statistically significant is <0.05. VAS: Visual Analogue Scale; ESR: erythrocyte sedimentation rate; CRP: C-reactive protein

Variable	N=126					
	RA=86	SpA=40	Test used	Test statistic		p-value
Age ^δ^	59.2±13.1	49.4±11.5	t-test	4.05	<0.001	
Females^¶^	77(89.53)	15(35.5)	Chi-square	37.5	<0.001	
Males^¶^	9(10.47)	25(64.5)	-	-	-	
Illiterate^¶^	49(56.97)	12.5(5)	Chi-square	23.7	<0.001	
Primary study^¶^	13(15.11)	9(22.5)	-	-	-	
Secondary school^¶^	16(18.60)	20(50)	-	-	-	
Higher education^¶^	8(9.30)	6(15)	-	-	-	
Rural^¶^	22(25.85)	6(15)	Chi-square	1.77	0.184	
Urban^¶^	64(74.41)	34(85)	-	-	-	
BMI^δ^	26.5±4.97	26.0±2.69	t-test	5.63	0.575	
High blood pressure^¶^	20(23.25)	3(7.5)	Chi-square	4.54	0.033	
Diabetes^¶^	11(12.7)	0(0)	Chi-square	5.61	0.018	
Dyslipidemia^¶^	16(18.60)	0(0)	Chi-square	8.52	0.004	
Thyroid disorder ^¶^	8(9.30)	1(2.5)	Chi-square	1.90	0.168	
Uveitis^¶^	0(0)	11(27.5)	Chi-square	25.90	<0.001	
Smoking^¶^	2(2.32)	2(5)	Chi-square	0.63	0.425	
Total knee prosthesis^¶^	2(2.32)	0(0)	Chi-square	0.94	0.331	
Total hip prosthesis^¶^	4(4.65)	7.5(3)	Chi-square	0.42	0.516	
Psoriasis^¶^	0(0)	1(2.5)	Chi-square	2.17	0.141	
Chronic inflammatory bowel disease^¶^	0(0)	1(2.5)	Chi-square	2.17	0.141	
History of depression^¶^	5(5.81)	0(0)	Chi-square	2.42	0.120	
Chronic kidney disease^ ¶^	17(19.76)	4(10)	Chi-square	2.97	0.562	
Heart disease^¶^	6(6.97)	0(0)	Chi-square	2.93	0.087	
VAS^δ^	3.4±1.5	1.7±1.1	t-test	6.09	<0.001	
ESR in mm/h^§^	25(10.5 ;38.5)	16(10.5 ;37)	Mann-Whitney U	1211	0.292	
CRP in mg/L^§^	5.12(1.7 ;11)	3.1(1.55 ;8.05)	Mann-Whitney U	1577	0.453	
Disease duration^δ^	17.5±9.85	16.2±8.47	Mann-Whitney U	1582	0.464	
Delay between symptom onset and diagnosis (years)^§^	1(1 ;4)	2(1 ;6)	Mann-Whitney U	1460	0.159	
Duration of exposure to biological therapy^§^	5(2 ;9)	6(2 ;10)	Mann-Whitney U	1690	0.559	
Anti-TNF^¶^	33(38.4)	39(97.5)	Chi-square	12.30	0.007	
Etanercept^¶^	18(20.9)	8(20)	-	-	-	
Adalimumab^¶^	8(9.3)	13(32.5)	-	-	-	
Infliximab^¶^	6(7)	8(20)	-	-	-	
Golimumab^¶^	1(1.2)	10(25)	-	-	-	
Rituximab^¶^	23(26.7)	-	-	-	-	
Tocilizumab^¶^	28(32.6)	-	-	-	-	
Secukinumab^¶^	-	1(2.5)	-	-	-	
Tofacitinib^¶^	2(2.3)	-	-	-	-	

Regarding disease activity, RA patients exhibited a mean DAS28-CRP of 3.27 ± 1.49, together with a median CDAI of 10 (4-16) and SDAI of 10.1 (4.6-17), indicating predominantly low to moderate disease activity. The median HAQ score of 0.70 (0.40-1.17) reflected a low functional impairment. In SpA patients, median ASDAS-CRP was 1.30 (1-1.71), BASDAI was 1.10 (0.77-2.10), and BASFI was 1.35 (0.87-2.40), consistent with low disease activity and relatively preserved functional status (Table 2).

**Table 2:** Disease activity of our patients Continuous variables are expressed as mean ± SD (δ) or median (IQR) (§). DAS28-CRP (RA): Remission <2.6, Low 2.6–3.2, Moderate 3.2–5.1, High >5.1; CDAI (RA): Remission ≤2.8, Low 2.9–10, Moderate 10.1–22, High >22; SDAI (RA): Remission ≤3.3, Low 3.4–11, Moderate 11.1–26, High >26; ASDAS-CRP (SpA): Inactive <1.3, Low 1.3–2.1, High 2.1–3.5, Very High >3.5; BASDAI (SpA): Remission <2, Low 2–<4, Moderate 4–6, High >6; BASFI (SpA): Functional index, higher scores indicate greater impairment. DAS28 CRP: Disease Activity Score in 28 Joints using C-reactive Protein; CDAI: Clinical Disease Activity Index; SDAI: Score Disease Activity Index; HAQ: Health Assessment Questionnaire; ASDAS CRP: Ankylosing Spondylitis Disease Activity Score using C-reactive Protein; BASDAI: Bath Ankylosing Spondylitis Disease Activity Index; BASFI: Bath Ankylosing Spondylitis Functional Index; RA: rheumatoid arthritis; SpA: spondyloarthritis

Variable	N=126	
	RA=86	SpA=40
DAS28 CRP^δ^	3.27±1.49	
CDAI^§^	10(4; 16)	
SDAI^§^	10.10(4.59; 17)	
HAQ^§^	0.70(0.40; 1.17)	
ASDAS CRP^§^		1.30(1; 1.71)
BASDAI^§^		1.10(0.77:2.10)
BASFI^§^		1.35(0.87; 2.40)

Catastrophizing scores

Psychological assessment revealed higher rates of catastrophizing in RA compared with SpA (52.3% vs. 25%, p=0.004). RA patients also reported higher scores for rumination (p=0.003) and helplessness (p<0.001) (Table 3).

**Table 3:** PCS, GAD-7, PHQ-9 scores of ours patients Continuous variables are expressed as median (IQR) (§) and compared using the Mann-Whitney U test. Categorical variables (¶) are presented as n (%) and compared using the chi-square test. The p-value considered statistically significant is <0.05. PCS: Pain Catastrophizing Scale; GAD-7: Generalized Anxiety Disorder-7 questionnaire; PHQ-9: Patient Health Questionnaire; RA: rheumatoid arthritis; SpA: spondyloarthritis

Variable	N=126									
	RA=86	SpA=40	Test used		Test statistic					p-value
PCS^¶^	45(52.32)	10(25)	Chi-square		8.29					0.004
Under score Rumination^§^	11(6 ;13)	5(2 ;11.30)	Mann-Whitney U		1161					0.003
Under score magnification^§^	6(2 ;9)	4(1 ;7.25)	Mann-Whitney U		1368				0.064	
Under score helplessness^§^	13(7 ;17)	6(2 ;11.30)	Mann-Whitney U	957				<0.001		
GAD-7^¶^	67(77.90)	23(57.50)	Chi-square			5.57	0.018			
PHQ-9^¶^	72(83.72)	22(55)	Chi-square			11.9	<0.001			

Depression and anxiety

Anxiety was present in 77.9% of RA and 57.5% of SpA patients (p=0.018), while depression was significantly more frequent in RA (83.7% vs. 55%, p<0.001) (Table 3).

Factors associated with catastrophizing

In univariate analysis, several factors were significantly associated with a higher likelihood of catastrophizing (PCS >30), including female sex, which showed increased odds (OR 3.39, 95% CI 1.39-8.28, p=0.007), although this association did not remain significant after adjustment (OR 1.86, 95% CI 0.52-6.66, p=0.343). Age and disease duration were not associated with catastrophizing. Educational level showed a strong gradient effect: compared with illiterate patients, those with primary (OR 3.37, p=0.024), secondary (OR 2.78, p=0.022), and higher education (OR 9.43, p=0.006) had higher odds of catastrophizing. After adjustment, only higher education remained independently associated (OR 7.76, 95% CI 1.16-52.03, p=0.035). Disease phenotype also influenced catastrophizing: RA patients had higher odds compared with SpA in univariate analysis (OR 3.29, p=0.005), but this association was no longer significant in multivariate models (OR 0.69, p=0.580). Pain intensity (VAS) was inversely associated with catastrophizing, both in univariate (OR 0.59, p<0.001) and multivariate analysis (OR 0.52, p<0.001), suggesting that patients with lower pain scores paradoxically reported higher levels of catastrophizing. Anxiety (GAD-7 ≥5) and depression (PHQ-9 ≥5) showed the strongest associations, with anxiety significant in both univariate (OR 7.75, p<0.001) and multivariate analysis (OR 6.87, p=0.004), while depression was significant only in univariate analysis (OR 11.97, p<0.001) and lost significance after adjustment (OR 4.02, p=0.055). Duration of biologic therapy was included in the multivariate analysis but was not significantly associated with catastrophizing (Table 4).

**Table 4:** Univariate and multivariate analysis of the factors associated with catastrophism in our population The p-value considered statistically significant is <0.05. F: female; M: male; RA: rheumatoid arthritis; SpA: spondyloarthritis; VAS: visual Analog Scale; GAD-7: Generalized Anxiety Disorder-7 questionnaire; PHQ-9: Patient Health Questionnaire-9

Catastrophism	Predictor	Univariate OR (95% CI)	p-value	Multivariate OR (95% CI)	p-value
Patients with a Pain Catastrophizing Scale (PCS) score >30 vs. <30	Sex (F vs. M)	3.39 (1.39–8.28)	0.007	1.86 (0.52–6.66)	0.343
	Age (years)	0.98 (0.95–1.01)	0.175	1.01 (0.96–1.05)	0.757
	Education (ref = illiterate)				
	Primary	3.37 (1.18–9.63)	0.024	1.97 (0.53–7.30)	0.311
	Secondary	2.78 (1.16–6.66)	0.022	1.42 (0.42–4.83)	0.571
	Higher	9.43 (1.92–46.41)	0.006	7.76 (1.16–52.03)	0.035
	RA vs. SpA (1 vs. 0)	3.29 (1.43–7.56)	0.005	0.69 (0.19–2.53)	0.58
	Disease duration (years)	0.99 (0.96–1.03)	0.637	1.00 (0.95–1.06)	0.961
	Pain VAS	0.59 (0.45–0.76)	<0.001	0.52 (0.36–0.76)	<0.001
	Biotherapy duration (years)	1.00 (0.93–1.08)	0.977	0.96 (0.86–1.07)	0.433
	GAD-7 (B vs. A)	7.75 (2.76–21.75)	<0.001	6.87 (1.82–25.93)	0.004
	PHQ-9 (B vs. A)	11.97 (3.41–42.03)	<0.001	4.02 (0.97–16.65)	0.055

## Discussion

In our cohort of 126 patients, we observed a high prevalence of catastrophizing, particularly among RA patients (52.3%) compared to those with SpA (25%). Subscore analysis revealed significantly higher levels of rumination and helplessness in RA patients, while magnification did not differ significantly. Furthermore, anxiety and depression were highly prevalent, with 77.9% and 83.7% of RA patients meeting thresholds on the GAD-7 and PHQ-9, respectively, compared to 57.5% and 55% of SpA patients. These results underscore the close interplay between psychological distress and catastrophizing in chronic inflammatory rheumatic diseases.

Our findings align with those of Penhoat et al., who reported that nearly one-fourth of RA and SpA patients on biotherapy had high PCS scores (≥30), with helplessness contributing most to elevated scores in SpA [19]. Interestingly, in our study, catastrophizing was more frequent in RA, contrasting with their observation of slightly higher PCS scores in SpA. This discrepancy may be attributable to differences in sociodemographic context, including educational levels and gender distribution, which were significant predictors in our analysis.

We observed that pain catastrophizing was significantly higher in RA compared with SpA patients, with 52.3% of RA patients and 25% of SpA patients exhibiting high PCS scores. These findings are in line with the Moroccan study by Khazzani et al., which reported a PCS ≥30 in 47.8% of their cohort, although their population included a broader range of rheumatic conditions (RA 54.3%, SpA 38.4%, and undifferentiated chronic inflammatory rheumatism 7.2%) [20]. Notably, our RA patients had markedly higher PCS scores compared to their cohort, whereas our SpA patients had lower scores, highlighting potential differences in psychosocial perception, disease chronicity, or healthcare access between the two populations. Regarding psychological factors, both studies consistently demonstrated that anxiety and depression were strongly associated with catastrophizing. In our multivariate analysis, GAD-7 and PHQ-9 remained the only independent predictors of high PCS, mirroring the findings of Khazzani et al., who also identified these psychological variables as the main determinants of catastrophizing.

The work of Putra et al. on the social construct of masculinity describes how traditional masculine norms, such as stoicism, self-reliance, and the avoidance of emotional expression, socialize men to minimize and underreport vulnerability, pain, and emotional distress [21]. In the context of our study, it is highly plausible that these norms influence the self-reporting on the PCS. Men might be less likely to endorse items related to helplessness ("There's nothing I can do to reduce the intensity of the pain") or rumination, not because they do not experience these feelings, but because acknowledging them violates internalized masculine ideals. Therefore, the initial univariate association may not reflect a greater propensity for women to catastrophize but rather a greater propensity for men to underreport catastrophic thinking due to social desirability bias and gendered coping mechanisms. This creates a measurement bias that the multivariate model helps to uncover by controlling for other variables.

Additionally, the older age of the RA population and the presence of cardiovascular comorbidities may further exacerbate the psychological burden, as older adults often face unique challenges such as social isolation and functional decline. These factors may collectively explain the disparity in catastrophizing levels between RA and SpA patients.

Finally, our study highlights the paradoxical negative association between pain VAS and catastrophizing, suggesting that catastrophizing may reflect anticipatory cognitive-emotional patterns rather than direct pain intensity.

Despite the use of biologic therapy, neither the duration of treatment nor the route of administration significantly influenced catastrophizing scores. This suggests that while biologic therapy effectively controls disease activity, it may not adequately address the psychological dimensions of chronic pain. Therefore, integrated approaches that combine pharmacologic treatment with psychological interventions, such as cognitive-behavioral therapy or mindfulness-based stress reduction, may be necessary to address catastrophizing and improve overall outcomes.

Limitations

Our study has several limitations. First, the cross-sectional design precludes causal inferences, and longitudinal studies are needed to explore the temporal relationship between catastrophizing and disease outcomes. Second, the limited geographic scope of our survey may affect the generalizability of the results to other populations. Third, the absence of a comparison group receiving conventional background therapy limits our ability to assess the specific impact of biologic therapy on catastrophizing. Fourth, the reliance on self-reported measures may introduce bias, particularly in the assessment of psychological variables. Finally, potential unmeasured confounding factors, such as pain chronicity and fibromyalgia, were not assessed. Additionally, recruitment limited to patients attending infusion or prescription renewal visits may over-represent patients with stable disease and under-represent those on subcutaneous biologics or experiencing active flares, which could introduce selection bias. While treatment duration was recorded, it was not significantly associated with catastrophizing in our analyses, representing a limitation in assessing its potential independent effect. Future studies should address these aspects to improve the generalizability and validity of the findings.

## Conclusions

Despite these limitations, our study demonstrates that catastrophizing is highly prevalent among RA and SpA patients receiving biologic therapy and is strongly associated with anxiety and depression. These findings underscore the importance of recognizing catastrophizing as an independent psychosocial dimension that persists even in patients with well-controlled disease activity. Routine screening for catastrophizing and psychological comorbidities should therefore be incorporated into clinical practice to better identify high-risk patients and guide holistic management. Integrating targeted psychological interventions, such as cognitive-behavioral therapy, pain education, or mindfulness-based strategies, alongside pharmacologic treatment may help reduce catastrophizing and ultimately improve functional outcomes and quality of life. Future longitudinal studies are needed to further clarify causal pathways and to assess whether combined psychological and pharmacologic approaches can modify the trajectory of catastrophizing in chronic inflammatory rheumatic diseases.

## Figures and Tables

**Figure 1:**
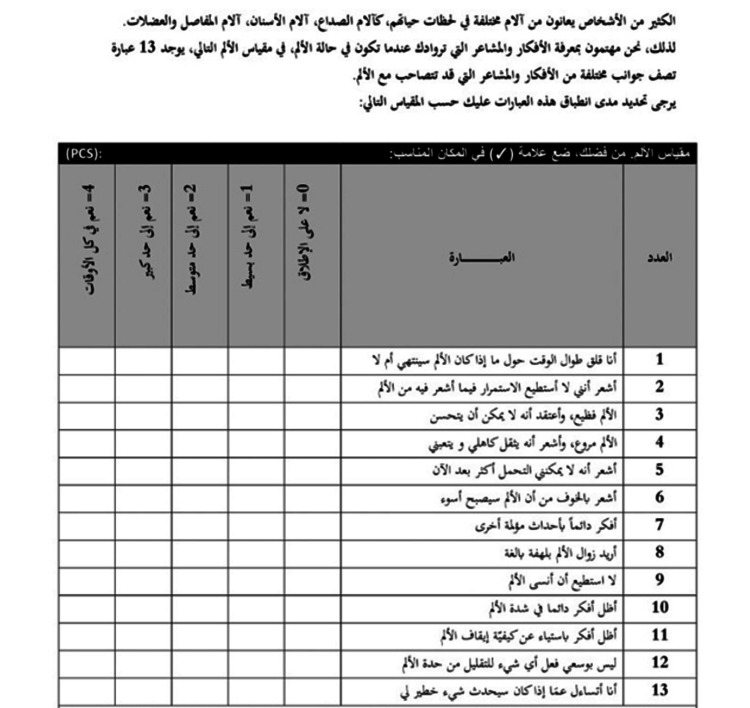
The Arabic version of the Pain Catastrophizing Scale (PCS) [15]

**Figure 2:**
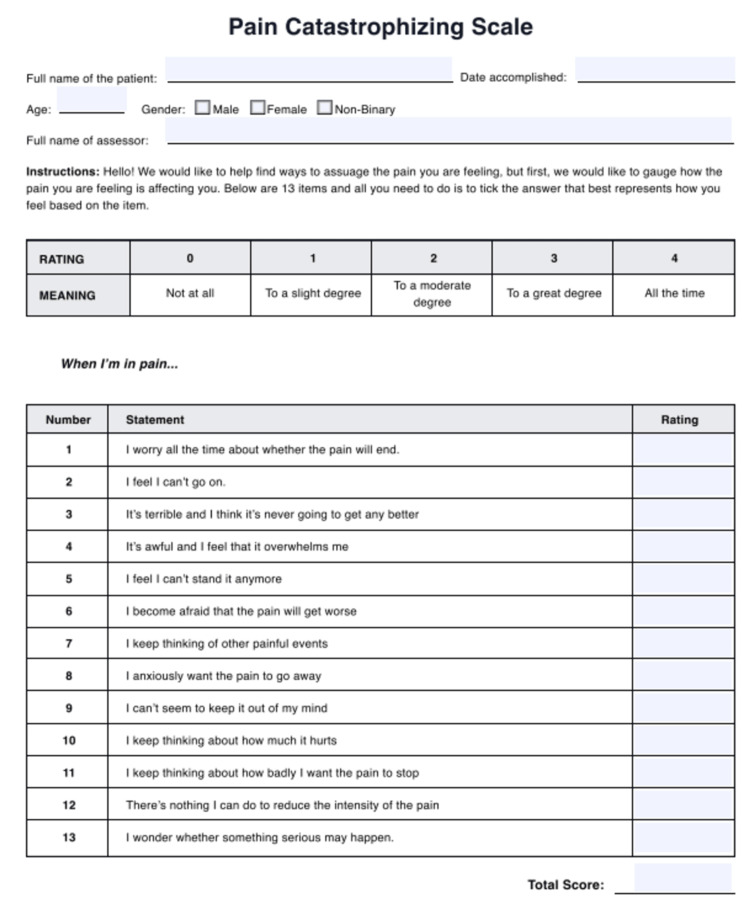
English translation of Pain Catastrophizing Scale (PCS) score [15]

**Figure 3:**
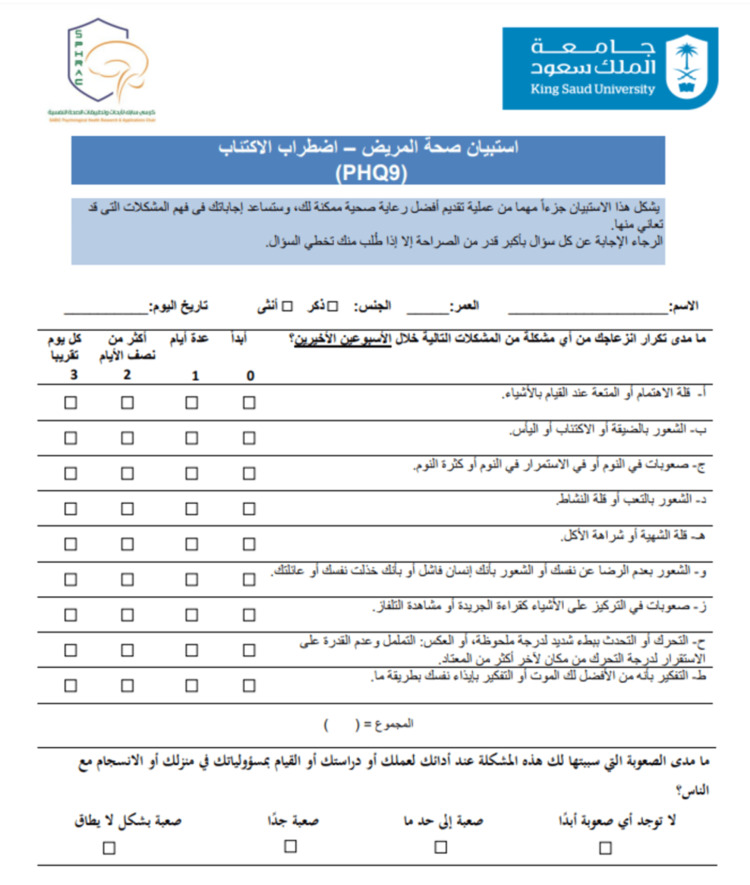
The Arabic version of the Patient Health Questionnaire-9 (PHQ-9) [16, 17]

**Figure 4:**
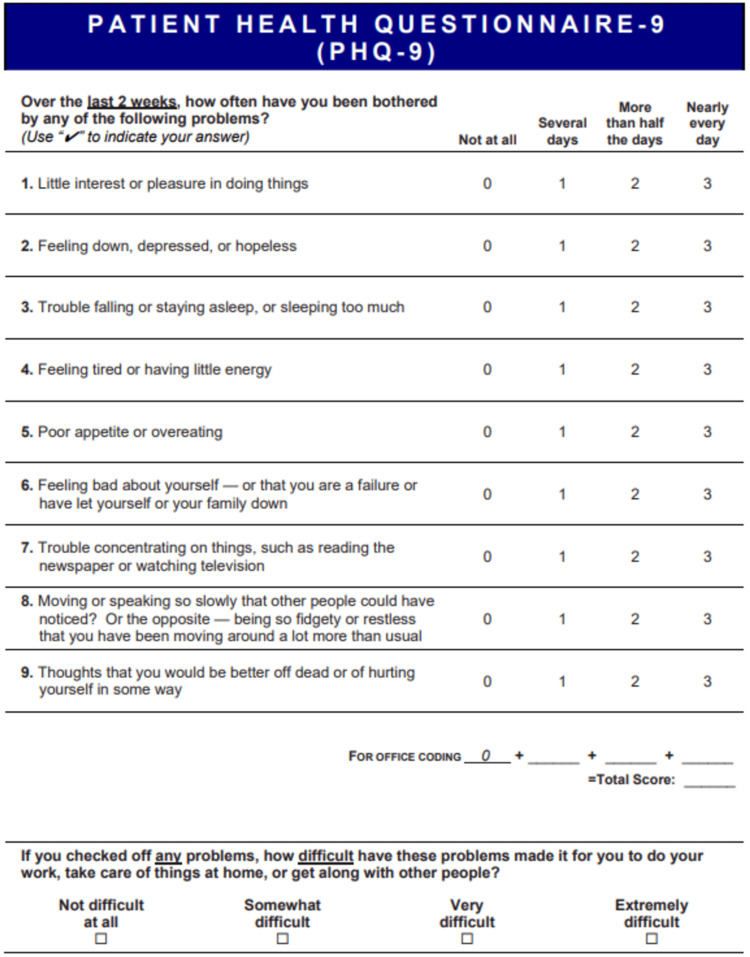
English translation of the Patient Health Questionnaire-9 (PHQ-9) score [16, 17]

**Figure 5:**
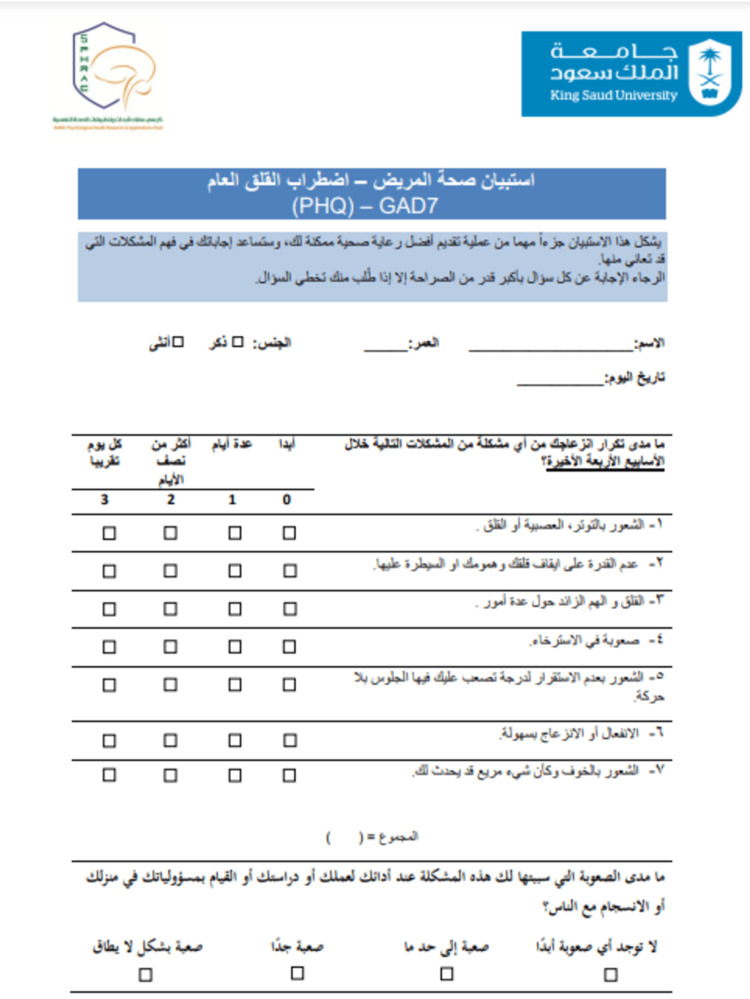
The Arabic version of the Generalized Anxiety Disorder-7 (GAD-7) [17]

**Figure 6:**
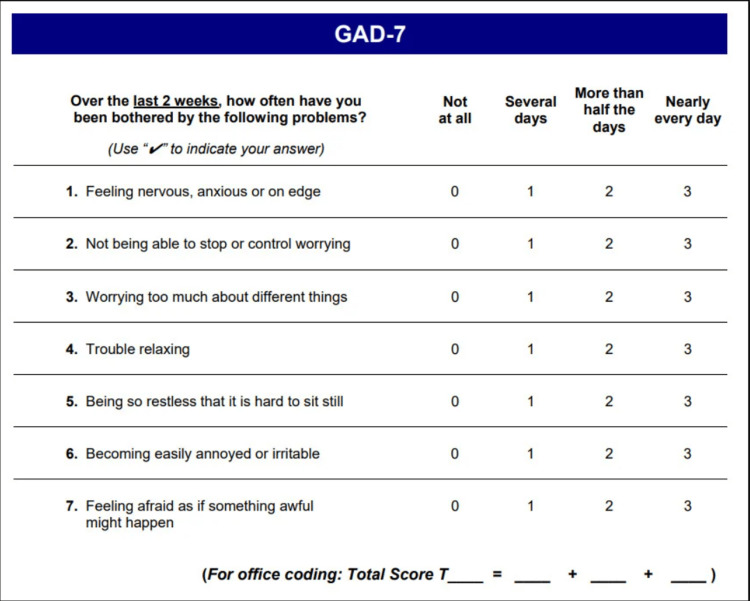
English translation of Generalized Anxiety Disorder-7 (GAD-7) score [17]

## Appendices

Appendix A 
